# Conventional pulse transit times as markers of blood pressure changes in humans

**DOI:** 10.1038/s41598-020-73143-8

**Published:** 2020-10-02

**Authors:** Robert C. Block, Mohammad Yavarimanesh, Keerthana Natarajan, Andrew Carek, Azin Mousavi, Anand Chandrasekhar, Chang-Sei Kim, Junxi Zhu, Giovanni Schifitto, Lalit K. Mestha, Omer T. Inan, Jin-Oh Hahn, Ramakrishna Mukkamala

**Affiliations:** 1 Department of Public Health Sciences, School of Medicine and Dentistry, University of Rochester, Rochester, NY USA; 2 Department of Electrical and Computer Engineering, Michigan State University, East Lansing, MI USA; 3 School of Electrical and Computer Engineering, Georgia Institute of Technology, Atlanta, GA USA; 4 Department of Mechanical Engineering, University of Maryland, College Park, MD USA; 5 School of Mechanical Engineering, Chonnam National University, Gwangju, Korea; 6 Departments of Neurology and Imaging, School of Medicine and Dentistry, University of Rochester, Rochester, NY USA; 7 Department of Electrical Engineering, University of Texas, Arlington, TX USA; 8 Department of Bioengineering and Anesthesiology and Perioperative Medicine, University of Pittsburgh, 302 Benedum Hall, 3700 O’Hara Street, Pittsburgh, PA 15261 USA

**Keywords:** Cardiology, Risk factors

## Abstract

Pulse transit time (PTT) represents a potential approach for cuff-less blood pressure (BP) monitoring. Conventionally, PTT is determined by (1) measuring (a) ECG and ear, finger, or toe PPG waveforms or (b) two of these PPG waveforms and (2) detecting the time delay between the waveforms. The conventional PTTs (cPTTs) were compared in terms of correlation with BP in humans. Thirty-two volunteers [50% female; 52 (17) (mean (SD)) years; 25% hypertensive] were studied. The four waveforms and manual cuff BP were recorded before and after slow breathing, mental arithmetic, cold pressor, and sublingual nitroglycerin. Six cPTTs were detected as the time delays between the ECG R-wave and ear PPG foot, R-wave and finger PPG foot [finger pulse arrival time (PAT)], R-wave and toe PPG foot (toe PAT), ear and finger PPG feet, ear and toe PPG feet, and finger and toe PPG feet. These time delays were also detected via PPG peaks. The best correlation by a substantial extent was between toe PAT via the PPG foot and systolic BP [− 0.63 ± 0.05 (mean ± SE); p < 0.001 via one-way ANOVA]. Toe PAT is superior to other cPTTs including the popular finger PAT as a marker of changes in BP and systolic BP in particular.

## Introduction

Current blood pressure (BP) measurement devices employ an inflatable cuff and thus cannot be used anytime or anywhere to manage hypertension. Pulse transit time (PTT) varies inversely with BP in a person due to the physical properties of arteries and can be obtained without a cuff. As a result, PTT is being widely pursued for cuff-less BP measurement^1,2^.

Conventionally, a surrogate of PTT is obtained by using two transducers amongst ECG electrodes and ear, finger, and toe photo-plethysmography (PPG) sensors due to their simplicity and noise robustness and detecting the relative timing between the pair of acquired waveforms^1^. The most popular of these conventional PTTs (cPTTs) has been the time delay between the R-wave of the ECG waveform and the ensuing foot or peak of the finger PPG waveform^1^. This time delay is in fact technically a pulse arrival time (PAT), which includes the pre-ejection period due to the use of the ECG waveform, rather than a true PTT and is referred to as finger PAT here. Similarly, the time delay between a pair of the PPG waveforms is technically a difference between two PTTs and is referred to as dPTT here. For example, the time delay between ear and toe PPG waveforms is called ear-toe dPTT, because it represents the difference between the PTT from the aortic arch to the toe and the PTT from the aortic arch to the ear. Note that the term cPTT therefore denotes a PAT or a dPTT henceforth rather than a true PTT.

Numerous studies have reported that the cPTTs can show inverse correlation with systolic and/or diastolic BP^1,3–10^. However, these studies have often been limited in terms of subjects or BP-varying interventions, so their results may not be generalizable. While studies have examined the PTT-BP relationship under challenging interventions, they have been confined to animals^8^, few healthy humans^9^, or critically ill patients^5–7,10^ who are often hypotensive and thus not reflective of the hypertension management population. Larger studies of normotensive and hypertensive humans have been conducted, but they have mainly involved few or simple BP interventions^1,3,4^. Often times, only exercise has been invoked wherein finger PAT is already known to decline with the parallel increases in systolic and diastolic BP^1^. Furthermore, the previous efforts have typically been limited to study of only finger PAT.

In this study, we compared the cPTTs as markers of BP changes under a battery of nontrivial BP-varying interventions in a relatively large number of normotensive and hypertensive humans.

## Methods

### Data collection

We collected physiologic data from human subjects under a protocol approved by, and in accordance with the relevant guidelines and regulations of, the Institutional Review Boards of University of Rochester and Michigan State University. All subjects gave written, informed consent prior to their participation in the study.

We studied the subjects at the University of Rochester’s General Clinical Research Center. The inclusion criteria were adults with self-reported capability of moderate exercise. The exclusion criteria included (a) cardiovascular disease exclusive of hypertension that was diagnosed earlier or identified upon physical exam or 12-lead ECG during a screening visit; (b) inter-arm mean BP difference > 10 mmHg during the screening visit; (c) systolic BP < 100 mmHg during this visit; (d) pregnancy; or (e) history of drug abuse or high alcohol consumption rate. A main purpose of the exclusion criteria was to mitigate potential adverse effects of the BP-varying interventions of the study. We enrolled 44 different subjects.

**Figure 1:**
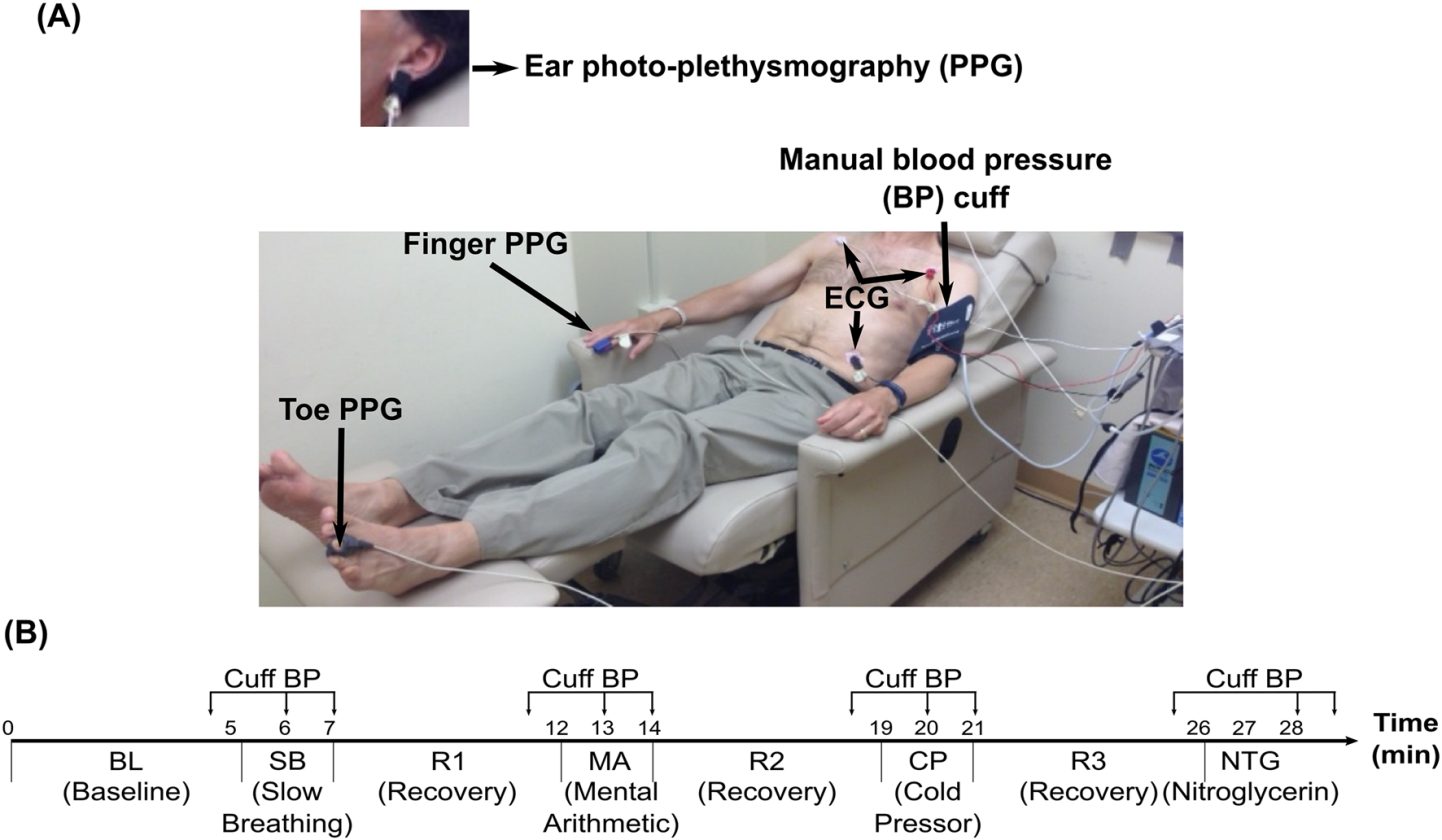
Data collection for comparing conventional pulse transit times (cPTTs) as markers of blood pressure (BP) changes in humans. The (**A**) sensors and (**B**) BP-varying interventions were employed in the reclining volunteer.

As shown in Fig. 1A, we placed sensors on each subject as she/he reclined on a chair. The sensors included electrode patches on the chest to measure an ECG waveform (ECG100C, Biopac, USA); transmission-mode PPG clip or soft sensors (8000 series, Nonin Medical, USA) on the earlobe, right fingertip, and toe to measure three PPG waveforms (PPG100C, Biopac); and an inflatable cuff over the left brachial artery to measure BP (Tycos TR-1, Welch Allyn, USA) via manual auscultation performed by a physician (R. C. B.). We also positioned standard impedance cardiography (ICG) electrodes (NICO100C customized with 4 mA current, Biopac) but found the ICG waveform quality to be poor and aborted this measurement well before study completion. We recorded the waveforms continuously at a sampling rate of 1 kHz using a single data acquisition system (MP150, Biopac). Hence, the waveforms were temporally synchronized.

As shown in Fig. 1B, we instructed each subject to perform a battery of interventions following a baseline period to change BP. The interventions comprised slow breathing (6 cycles/min for 2-min) to reduce BP^11^, mental arithmetic (successively adding digits of a 3-digit number and then adding the sum to the original number for 2-min) to increase BP^12^, a cold pressor test (foot immersed in 4 °C water for 2-min) to increase BP^13^, and sublingual nitroglycerin (0.4 mg tablet under the tongue) to reduce systolic BP but not alter diastolic BP^14^. Five-minute recovery periods occurred between the interventions. For safety considerations, we did not employ nitroglycerin in those subjects with pre-intervention systolic BP < 110 mmHg (19 subjects). As also shown in Fig. 1B, we made manual cuff measurements of systolic/diastolic BP during baseline, each intervention (at the end and typically the middle), and each recovery period (at the end) for a total of up to twelve sets of four waveforms and BP readings during up to eight different conditions per subject.

### Data analysis

**Figure 2:**
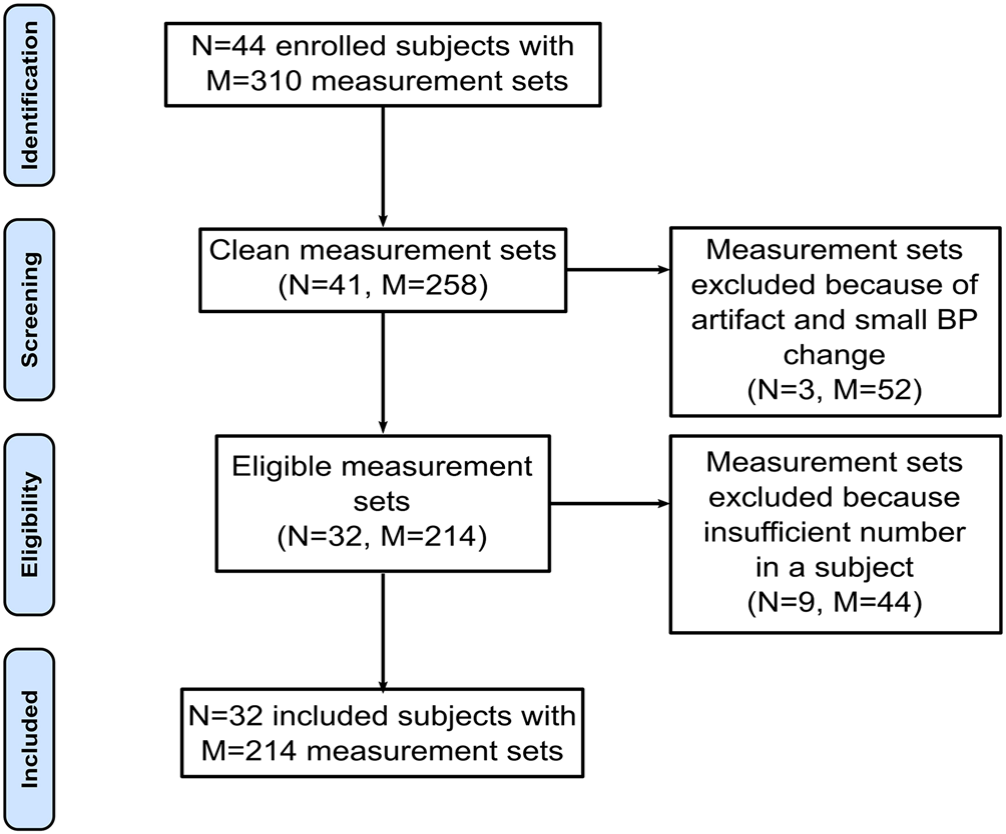
Data exclusion criteria with number of included and excluded subjects and measurement sets. A measurement set comprises the ECG waveform, ear, finger, and toe photo-plethysmography (PPG) waveforms, and manual cuff BP for a subject and condition (see Fig. 1B). The criteria were strict to ensure a valid comparison of cPTTs as markers of BP changes.

As shown in Fig. 2, we applied strict data exclusion criteria to ensure a meaningful, apples-to-apples comparison of the intra-subject correlations between each cPTT detected from pairs of the waveforms and each BP over the multiple conditions. We visually screened the waveform segments within ± 30 s of each BP measurement for artifact. We selected a > 7 s sub-segment for which all four waveforms showed minimal artifact. If there was no such sub-segment, we excluded the entire waveform-BP set from further analysis. Our rationale for such exclusion was as follows. In most cases of artifact, not all four waveforms were contaminated. If we kept the measurement set, then the cPTTs detected from the artifact-corrupted waveform(s) would be unfairly handicapped. If we discarded only the noisy waveform(s) in the set, then the cPTTs would not be compared using the same data (e.g., the correlations would be computed based on a different number of data points). If there were two sets of artifact-free waveform sub-segments and BP readings for a condition, we excluded the set with the smaller BP change in order to maximize the intra-subject BP variations. We excluded entire subject records with less than five measurement sets in subjects without nitroglycerin and less than six measurement sets or six measurement sets without three or more interventions in subjects with all four interventions. Our reasoning was that if we kept subjects with few measurement sets, then the computed correlation between cPTT and BP would be misleading (e.g., two data points always yield unity correlation). Hence, a significant fraction of the data were excluded for a valid comparison, but the quality of data from the simple, robust sensors was actually much better.

**Figure 3:**
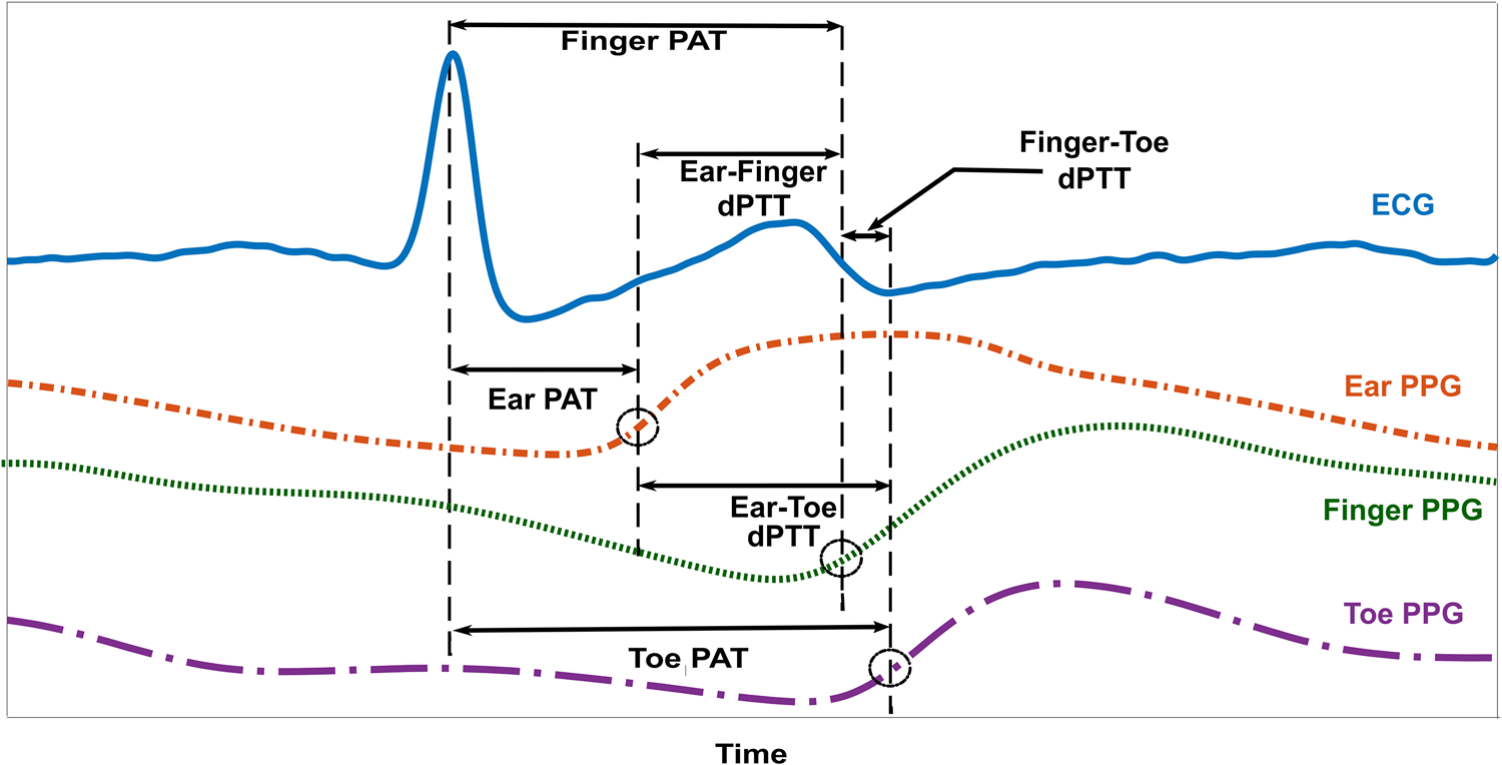
Data analysis for comparing cPTTs as markers of BP changes in humans. The six detected time delays include three pulse arrival times (PATs) and three differences between two PTTs (dPTTs). The time delays were averaged over multiple beats and compared in terms of tracking the intervention-induced BP changes via the intra-subject correlation coefficient.

We analyzed all included waveform sub-segments as follows. We first detected the R-waves of the ECG waveforms using the Pan-Thompkins algorithm. We then detected the peaks of each PPG waveform between successive R-waves. We next detected the feet of the waveforms between the R-waves and successive peaks using the intersecting tangent algorithm^1^. As shown in Fig. 3, we determined the cPTTs as the time delays (averaged over the sub-segment) between the ECG R-wave and ear PPG foot (ear PAT), ECG R-wave and finger PPG foot (finger PAT), ECG R-wave and toe PPG foot (toe PAT), ear and finger PPG feet (ear-finger dPTT), ear and toe PPG feet (ear-toe dPTT), and finger and toe PPG feet (finger-toe dPTT). For comparison, we also used the peaks instead of the feet to determine the six cPTTs.

We quantified the strength of the cPTT-BP relationship in each subject using the standard Pearson correlation coefficient. Another metric that has been previously employed for quantifying the PTT-BP relationship strength may be obtained by fitting a curve (e.g., BP = m × cPTT + b) through the cPTT and BP pairs of a subject and then computing the standard deviation (SD) of the fitting error in units of mmHg^1,2^. However, the correlation coefficient is preferred to this BP error SD for two reasons. One reason is that the correlation coefficient takes the sign of the relationship into account. That is, cPTT and BP should be negatively correlated. So, if cPTT and BP showed positive correlation in a subject, the correlation coefficient would penalize for this non-physiologic relationship through its sign. In contrast, the BP error SD is blind to the sign of the correlation. For example, if cPTT and BP showed strong, positive correlation in a subject, then the BP error SD would be small despite the non-physiologic relationship. The second reason is that the correlation coefficient takes the size of the BP variations within a subject into account. That is, the square of the correlation coefficient (a.k.a., R^2^) indicates the fraction of the total BP variance that is explained by a cPTT. In contrast, the BP error SD strongly depends on the intra-subject BP variation size. For example, if the intra-subject BP changes were small, then the BP error SD would be low even with no cPTT-BP correlation. (In such a case, the BP error SD would simply equal the BP SD.) For these reasons, the BP error SD was misleading here, so we only employed the correlation coefficient.

We arrived at one correlation coefficient for each cPTT and each BP per subject. We compared the mean correlation coefficients (i.e., the average of the correlation coefficients over the subjects) of the six cPTTs for each BP. Since there were more than two mean correlation coefficients, we conducted this comparison using one-way repeated measures ANOVA. When this test yielded p < 0.05, we performed pairwise comparisons using a Tukey test, which corrects for the multiple comparisons. We also performed paired t-tests when comparing two mean correlation coefficients.

## Results

As shown in Fig. 2, a total of 214 sets of cPTTs and manual cuff systolic/diastolic BP readings from 32 subjects [50% female; 52 (17) (mean (SD)] years of age; 166 (10) cm in height; 89 (34) kg in weight; 31% with smoking history; 9% with LDL cholesterol ≥ 190 mg/dL) were included. Eight subjects self-reported as hypertensive and were taking medications (e.g., Hydrochlorothiazide, Atorvastatin, Metoprolol, Lisinopril, Losartan, Aspirin, and Amlodipine).

**Figure 4:**
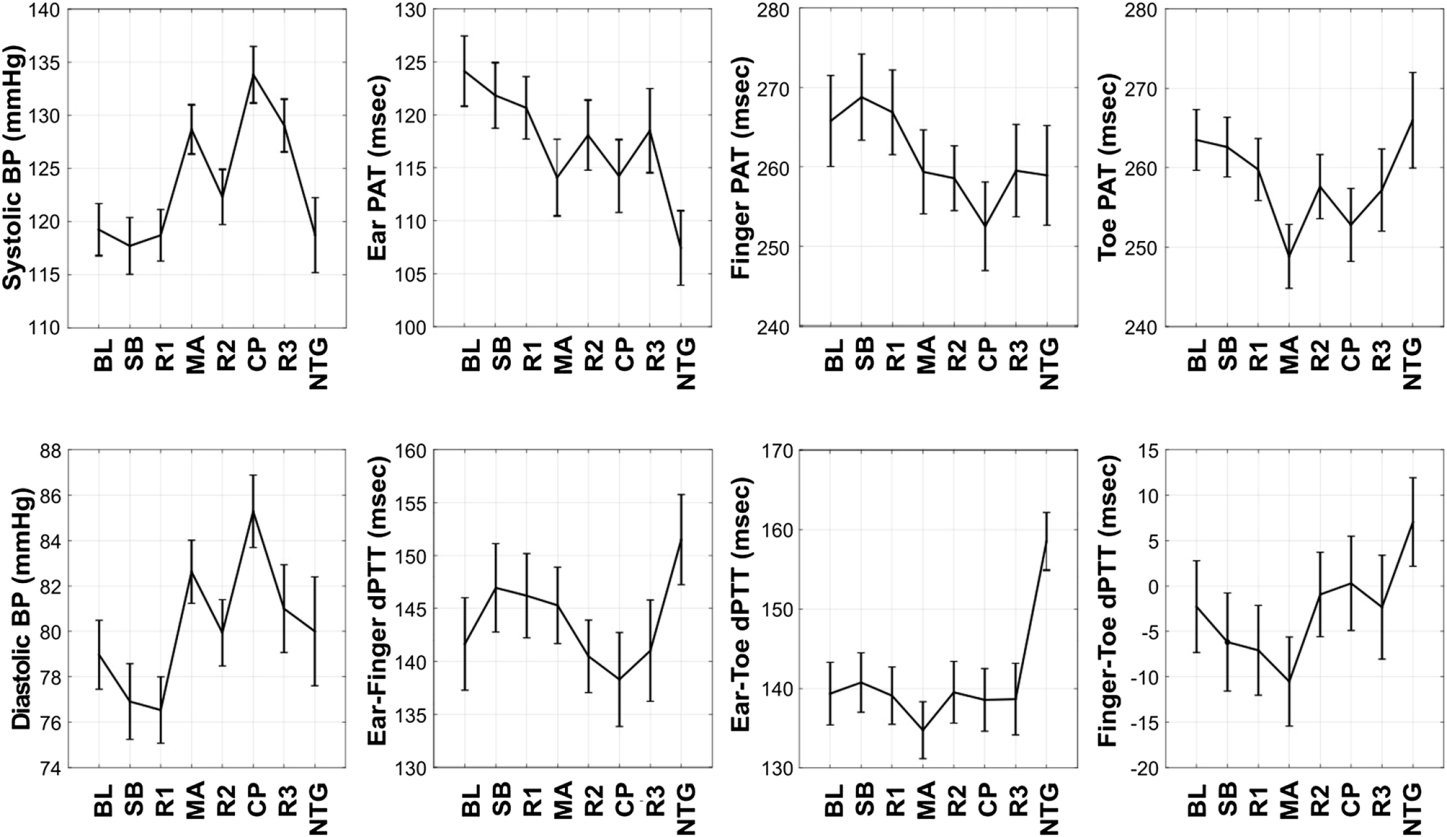
Mean (with SE) over the subjects (N = 32) of systolic and diastolic BP and the cPTTs for the baseline period, each intervention, and each recovery period (see Figs. 1 and 3 for definitions of interventions and cPTTs). The toe PAT trend appeared most inversely related to the systolic BP trend, whereas the toe PAT and finger PAT trends appeared most inversely related to the diastolic BP trend.

Figure 4 shows the mean (with SE) over the subjects of each cPTT shown in Fig. 3 and each BP for the baseline period, four interventions, and three recovery periods. (Note that we did not normalize each cPTT for wave travel distance per subject, as the height of the subjects varied little.) The baseline (BL) systolic and diastolic BP were 121 ± 3 and 79 ± 2 mmHg (where X ± Y denotes mean of X and SE of Y over the subjects here and henceforth). The subject cohort thus constituted mainly normotensives and controlled hypertensives. The baseline (BL) ear, finger, and toe PATs were 126 ± 4, 269 ± 6, and 266 ± 5 ms, respectively. While these values are consistent with previous data^1^, the comparable magnitudes of the finger and toe PATs may be in part due to a hydrostatic effect in the reclining subject (see Fig. 1A). In particular, the effective BP for finger PAT may be lower than that for toe PAT so as to increase finger PAT relative to toe PAT. Hence, BL ear-finger dPTT (which also equals finger PAT–ear PAT) and ear-toe dPTT (which also equals toe PAT–ear PAT) were comparable in magnitude, whereas BL finger-toe dPTT (which also equals toe PAT–finger PAT) was near 0 ms. Slow breathing (SB) caused little change in BP on average (see Fig. 4). As expected and on average, mental arithmetic (MA) and cold pressor (CP) increased systolic and diastolic BP, whereas nitroglycerin (NTG) reduced systolic BP but did not alter diastolic BP (see Fig. 4). Overall, the interventions caused systolic and diastolic BP to range respectively over 25 ± 1 and 15 ± 1 mmHg per subject (result not indicated in Fig. 4). Further, the correlation coefficient between systolic and diastolic BP was 0.49 ± 0.07. Hence, the two BP levels did not merely change in parallel. The range of the cPTTs were similar with an overall mean of 26 ± 3 ms per subject (result not indicated in Fig. 4). As can be seen in Fig. 4, the toe PAT trend showed the best inverse correlation with the systolic BP trend, while the toe PAT and finger PAT trends showed the best inverse correlation with the diastolic BP trend. The other cPTT trends did not appear to correlate well with either of the two BP trends.

**Figure 5:**
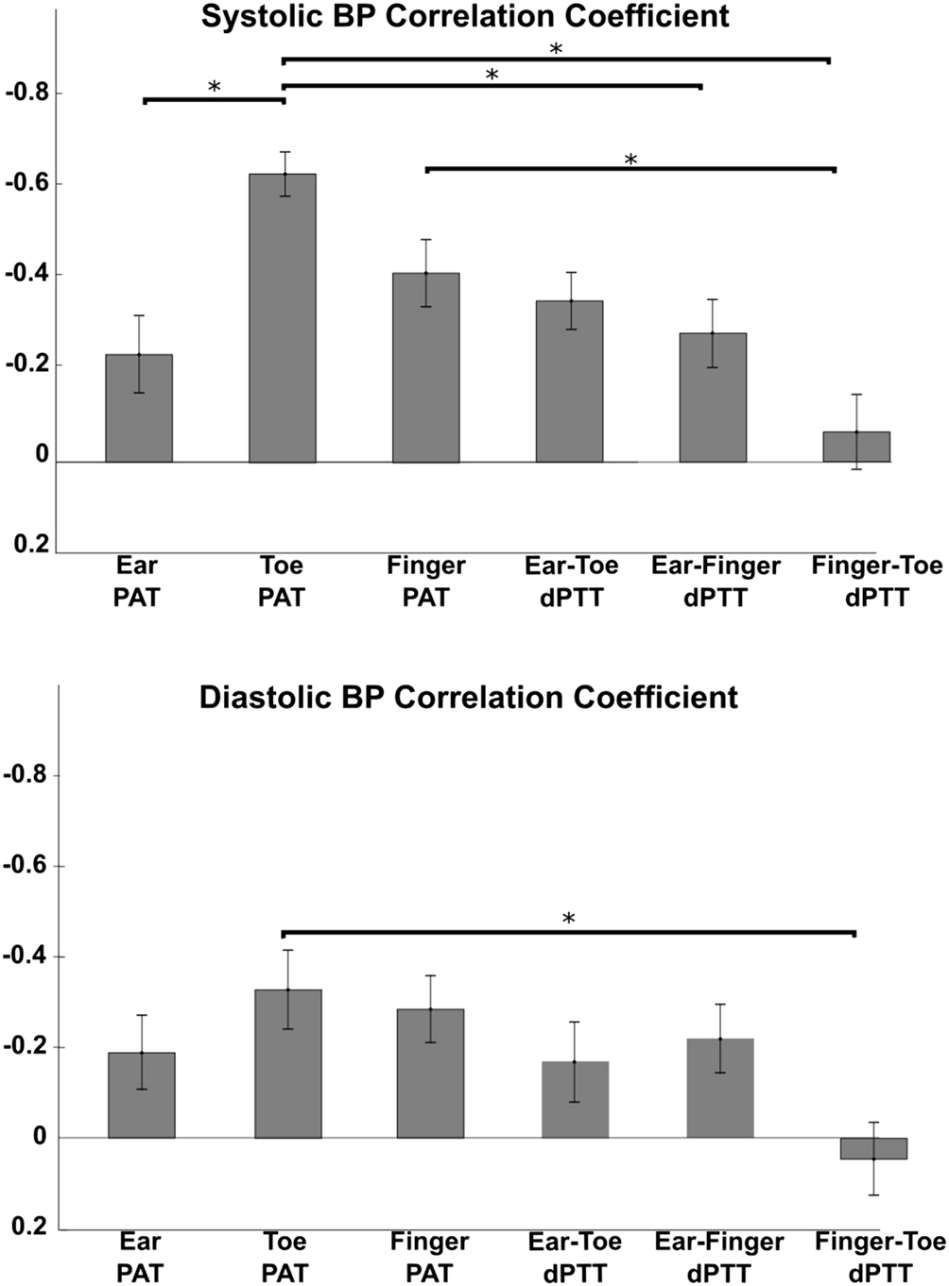
Mean (with SE) of the correlation coefficients between each cPTT and each BP over the subjects (see Figs. 1 and 3 for cPTT definitions). The correlation coefficients differed significantly for both systolic and diastolic BP according to one-way ANOVA. *Indicates significant pairwise differences based on a Tukey test. The best correlation by a substantial extent was between toe PAT and systolic BP.

Figure 5 shows the mean (with SE) of the correlation coefficients between each cPTT shown in Fig. 2 and each BP over the subjects as well as the results of one-way repeated measures ANOVA and the Tukey test. These correlation coefficients significantly differed for the two BP levels. Toe PAT tracked both BP levels best. The correlation coefficients were appreciably higher for systolic BP than diastolic BP. The correlation coefficient between toe PAT and systolic BP was − 0.63 ± 0.05, the only one above 0.5 in magnitude, and 54% higher in magnitude than the corresponding correlation coefficient for the popular finger PAT.

**Figure 6:**
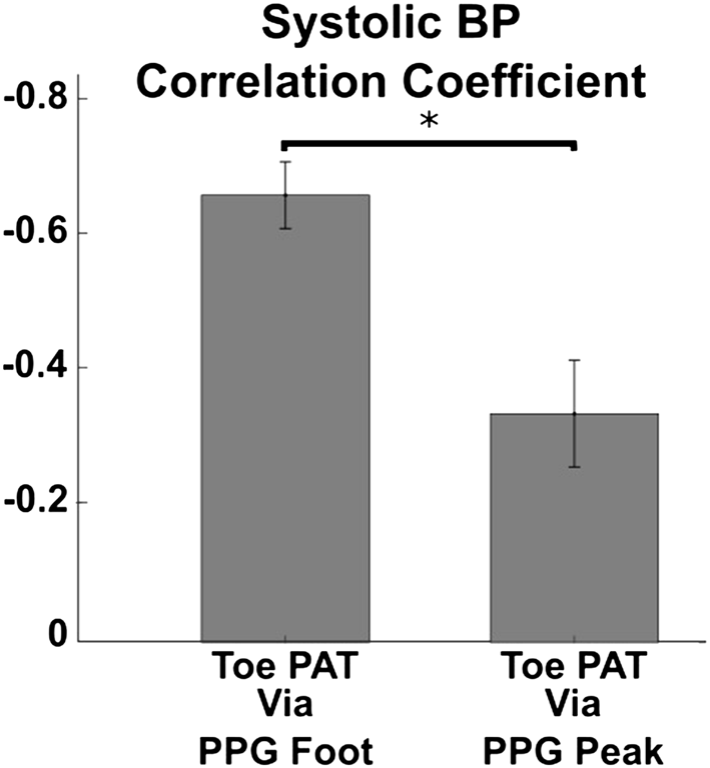
Mean (with SE) of the correlation coefficients between toe PAT detected via the PPG foot (see Fig. 3) and peak and systolic BP over the subjects. *Indicates significant difference based on a paired t-test. Toe PAT detected via the PPG foot afforded much better correlation.

The mean correlation coefficients between each cPTT detected via the PPG waveform peaks (instead of feet) and each BP over the subjects were all substantially lower than those shown in Fig. 5. Figure 6 shows an examplary comparison of the mean (with SE) of the correlation coefficients of toe PAT detected via the PPG waveform foot and peak and systolic BP over the subjects (p ≤ 0.001 as per a paired t-test).

**Figure 7:**
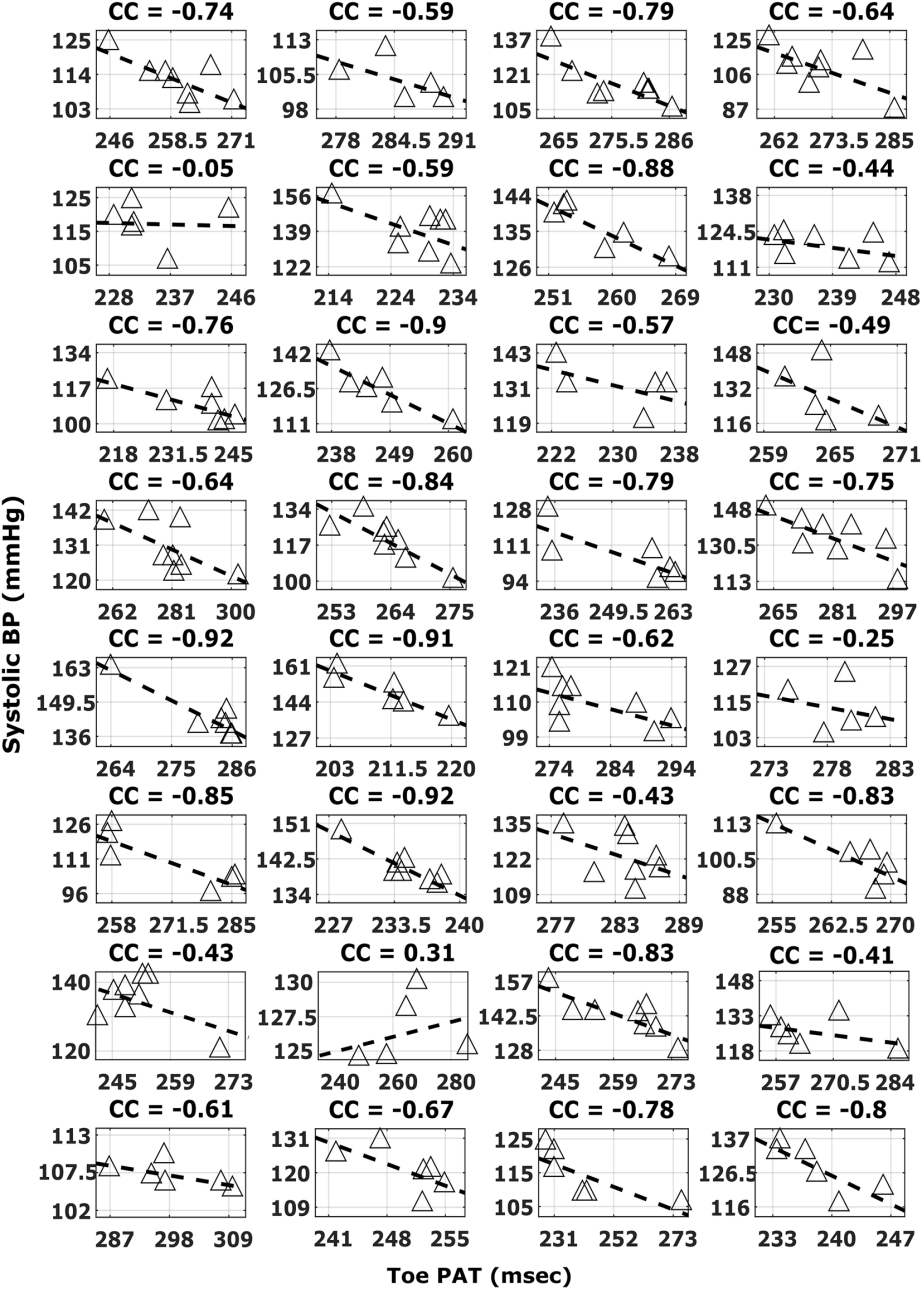
Subject-by-subject plots of systolic BP versus toe PAT detected via the PPG foot (see Fig. 3). Dashed lines are lines of best fit. The correlation coefficient (CC) varied per subject.

In sum, the best correlation by a substantial extent was between toe PAT detected via the PPG waveform foot and systolic BP (− 0.63 ± 0.05 correlation coefficient). Figure 7 shows subject-by-subject plots of this toe PAT versus systolic BP for all 32 subjects.

## Discussion

In this study, we compared cPTTs detected from ECG, ear, finger, and toe PPG waveforms in terms of tracking BP changes induced by interventions in humans. Our study may be notable relative to previous efforts in that it comprised (1) a relatively large number of human subjects (N = 32; see reference^1^ for N of similar studies) with appreciable diversity (25% hypertensive and average age of 52 ± 17 years); (2) acquisition of multiple cPTTs instead of just the popular finger PAT (see Figs. 1A and 3); (3) manual cuff BP measured by a physician rather than typically used but less accurate automatic cuff BP readings; and (4) a battery of interventions to change BP nontrivially instead of just typical exercise (see Figs. 1B and 4).

The most important of these strengths may be the interventions, which comprised slow breathing, mental arithmetic, a cold pressor test, and sublingual nitroglycerin. Although a previous study showed that two minutes of slow breathing reduces BP, especially in hypertensives^11^, this intervention had minimal impact on BP here (see Fig. 4). However, mental arithmetic increased systolic and diastolic BP via mainly an inotropic effect^12^; cold pressor increased systolic and diastolic BP via mainly vasoconstriction^13^; and nitroglycerin reduced systolic BP without changing diastolic BP via a vasodilatory effect^14^ (see Fig. 4). As a result, these interventions collectively caused systolic and diastolic BP to change appreciably (25 ± 1 and 15 ± 1 mmHg) and not merely in parallel (intra-subject correlation coefficient of 0.49 ± 0.07). In this way, we could not only determine the cPTT with the best association with BP but even determine the relative association with systolic and diastolic BP. Interestingly, the PPG amplitude increased with mental arithmetic and sublingual nitroglycerin and decreased with cold pressor (result not shown), suggesting that PPG amplitude may not readily indicate BP changes. The likely reason is that the amplitude of the PPG, which indicates blood volume, is approximately equal to the product of local arterial compliance and pulse pressure and that compliance changes with smooth muscle contraction (e.g., compliance decreases during cold pressor such that the PPG amplitude decreases despite the increase in pulse pressure shown in Fig. 4).

We found that toe PAT (the time delay between the ECG R-wave and the ensuing foot of the toe PPG waveform) was best in tracking the intervention-induced BP changes amongst six cPTTs (see Fig. 5). The next best cPTT as a marker of BP changes was the popular finger PAT (the analogous time delay via the finger PPG waveform). However, toe PAT afforded 54% better correlation with systolic BP than finger PAT (see Fig. 5).

Despite being detected at the level of diastole of the PPG waveform, toe PAT (and the other cPTTs) correlated much better with systolic BP than diastolic BP (see Fig. 5). One reason may be that toe PAT includes the pre-ejection period (PEP), which, like systolic BP, is partly determined by ventricular properties^1^. Another reason is that nitroglycerin decreased systolic BP, did not alter diastolic BP, and increased toe PAT via smooth muscle relaxation. Hence, toe PAT changed opposite to systolic BP, but not diastolic BP, during nitroglycerin (see Fig. 4). However, this result may be serendipitous, as the toe PAT increase during nitroglycerin may have been due to smooth muscle relaxation rather than the systolic BP decline. On the other hand, the correlations between each cPTT and BP were similar in the subjects who received nitroglycerin versus the subjects who did not receive the intervention (results not shown).

Detecting toe PAT (and the other cPTTs) using the PPG waveform peaks yielded substantially lower correlations with BP (see Fig. 6). This finding is not surprising, because the time delay between the foot and ensuing peak of the PPG waveform is largely determined by ventricular properties. In a recent study, we showed that PPG sensor contact pressure, which was controlled in the present study, impacts finger PAT detected via the PPG waveform peak twice as much as finger PAT detected via the PPG waveform foot^15^. Taken together, the pair of studies indicate that PTT should be detected specifically via the feet of PPG waveforms.

Hence, the best correlation by a substantial extent was between toe PAT detected via the PPG waveform foot and systolic BP. However, the correlation coefficient was only − 0.63 ± 0.05. These findings (see Figs. 4 and 5 in particular) may be the result of numerous, complicating factors, so a unifying interpretation may be impossible. Generally speaking, major factors limiting the correlation are that the PATs include PEP, which is again determined by ventricular properties rather than just BP, and that all cPTTs excluding toe PAT include substantial wave travel time through smaller, muscular arteries wherein smooth muscle contraction/relaxation can cause the time delay to vary independently of BP^14^. Another factor limiting the correlation for the dPTTs could be that detection of the PPG waveform feet is less robust than detection of the ECG waveform R-wave.

In contrast to many previous studies of finger PAT during exercise, our results indicate that finger PAT does not provide good correlation with systolic or diastolic BP. So, even though convenient devices can be developed to obtain finger PAT (e.g., smartwatch form factor), such efforts may not be worthwhile for cuff-less BP tracking. Our results indicate that it may make more sense to build devices to measure toe PAT. Note that building a convenient, portable system to measure toe PAT would be more challenging than finger PAT (as two recording devices may be needed). However, our study, instead, suggests that it may be necessary to obtain innovative PTTs to improve the correlation with BP via novel sensors and/or waveform detections. Innovative sensors may include ballistocardiography^16^, seismocardiography^17,18^, wrist bioimpedance^19^, ultrasound^20^, and radar sensors^21^. Unlike ECG and PPG waveforms, these sensors may afford true PTT rather than dPTT or PAT. However, they may be more complicated and/or less robust to artifact. Innovative waveform detections may include system identification methods to more robustly extract the PTTs from the entire waveforms rather than just their feet^22^ or to extract true PTT rather than dPTT from two distal waveforms^23^. However, advanced methods beyond waveform feet detection require much more proof.

Our study does have limitations. Firstly, while the number of subjects is relatively large compared to similar studies, this number is small compared to standardized protocols, and a quarter of the subjects were excluded for a meaningful, apples-to-apples comparison. Secondly, the interventions did change systolic and diastolic BP appreciably but not as much as we hoped. Thirdly, although 25% of the study cohort were self-reported hypertensives, these subjects had their BP under control on average. Fourthly, the impact of medications on the results was not assessed. Future studies of cPTTs should include more subjects, uncontrolled hypertensives, the same subjects with and without medications, and, if possible, interventions that produce more extensive BP changes. We do anticipate that such studies may reveal further limitations of cPTTs as markers of BP changes.

In conclusion, finger PAT and cPTTs detected via the PPG waveform peaks may not generally correlate well with BP in a person. Toe PAT may be a superior marker of changes in systolic BP in particular but perhaps not good enough. Innovations are needed in order to achieve cuff-less BP measurement via PTT.

## Data Availability

The raw data in this study will be made freely available at PhysioNet (www.physionet.org) upon completion of the NIH project. Until then, the data may be requested from R. M. (rmukkamala@pitt.edu).
